# The N-terminal polypeptide derived from viral macrophage inflammatory protein II reverses breast cancer epithelial-to-mesenchymal transition via a PDGFRα-dependent mechanism

**DOI:** 10.18632/oncotarget.16394

**Published:** 2017-03-21

**Authors:** Qing-Ling Yang, Ling-Yu Zhang, Hai-Feng Wang, Yu Li, Yue-Yue Wang, Tian-Tian Chen, Meng-Fen Dai, Hai-Hua Wu, Su-Lian Chen, Wen-Rui Wang, Qiong Wu, Chang-Jie Chen, Cong-Zhao Zhou

**Affiliations:** ^1^ Hefei National Laboratory for Physical Sciences at Microscale and the Innovation Center for Cell Signaling Network, School of Life Sciences, University of Science and Technology of China, Hefei, Anhui 233030, China; ^2^ Department of Biochemistry and Molecular Biology, Bengbu Medical College, Bengbu, Anhui 233030, China; ^3^ Clinical Testing and Diagnose Experimental Center of Bengbu Medical College, Bengbu, Anhui 233000, China; ^4^ Department of Biotechnology, Bengbu Medical College, Bengbu, Anhui 233030, China; ^5^ Department of Medical Oncology, First Affiliated Hospital of Bengbu Medical College, Bengbu, Anhui 233004, China

**Keywords:** breast cancer, paclitaxel, epithelial-mesenchymal transition, viral macrophage inflammatory protein II, PDGFRα

## Abstract

NT21MP, a 21-residue peptide derived from the viral macrophage inflammatory protein II, competed effectively with the natural ligand of CXC chemokine receptor 4 (CXCR4), stromal cell-derived factor 1-alpha, to induce apoptosis and inhibit growth in breast cancer. Its role in tumor epithelial-to-mesenchymal transition (EMT) regulation remains unknown. In this study, we evaluated the reversal of EMT upon NT21MP treatment and examined its role in the inhibition of EMT in breast cancer. The parental cells of breast cancer (SKBR-3 and MCF-7) and paclitaxel-resistant (SKBR-3 PR and MCF-7 PR) cells were studied *in vitro* and in combined immunodeficient mice. The mice injected with SKBR-3 PR cells were treated with NT21MP through the tail vein or intraperitoneally with paclitaxel or saline. Sections from tumors were evaluated for tumor weight and EMT markers based on Western blot. *In vitro*, the effects of NT21MP, CXCR4 and PDGFRα on tumor EMT were assessed by relative quantitative real-time reverse transcription–polymerase chain reaction, western blot and biological activity in breast cancer cell lines expressing high or low levels of CXCR4. Our results illustrated that NT21MP could reverse the phenotype of EMT in paclitaxel-resistant cells. Furthermore, we found that NT21MP governed PR-mediated EMT partly due to controlling platelet-derived growth factors A and B (PDGFA and PDGFB) and their receptor (PDGFRα). More importantly, NT21MP down-regulated AKT and ERK1/2 activity, which were activated by PDGFRα, and eventually reversed the EMT. Together, these results indicated that CXCR4 overexpression drives acquired paclitaxel resistance, partly by activating the PDGFA and PDGFB/PDGFRα autocrine signaling loops that activate AKT and ERK1/2. Inhibition of the oncogenic EMT process by targeting CXCR4/PDGFRα-mediated pathways using NT21MP may provide a novel therapeutic approach towards breast cancer.

## INTRODUCTION

In China, more than 1.6 million people are newly diagnosed with breast cancer and 1.2 million breast cancer deaths are expected to occur among women each year [1]. The American Cancer Society released the 2013 national cancer statistics report that ranked breast cancer as the highest incidence in women (29%) and the second highest death rate [2].

Paclitaxel (Taxol) is a powerful chemotherapeutic that has been used to treat ovarian, breast, lung, pancreatic, and other cancers [3, 4], yet its efficacy is limited due to chemoresistance. Paclitaxel resistance is associated with the acquisition of the epithelial-to-mesenchymal transition (EMT) [5]. Classical EMT-related signaling pathways, such as transforming growth factor-β (TGF-β), NF-κB-Snail, ErbB/EGF, and p38-MAPK, regulate the EMT in breast carcinoma [6, 7].

Crosstalk exists between the TGF-β and the G protein-coupled receptor CXCR4 pathways in liver tumors [8]. Signals from the microenvironment profoundly influence breast cancer maintenance and progression. CXCL12, also known as stromal cell–derived factor-1 (SDF-1α), binds to CXCR4, which is often overexpressed in breast cancer and has been correlated with poor clinical outcome [9, 10]. SDF-1α–CXCR4 signaling has been shown to play a key role in tumor growth, invasion, and angiogenesis [11–14]. The results of these studies indicate that SDF-1α overexpression in the tumor microenvironment may alter invasive capacity as well as the tumor-associated immune cells that are recruited to tumors. SDF-1α overexpression has been linked to increased metastasis and poor prognosis [15]. Targeting the SDF-1α–CXCR4 signaling pathway has also been studied in breast cancer treatment [16, 17]. High CXCR4 overexpression from breast cancer patients receiving neoadjuvant chemotherapy was predictive of poorer prognosis [18]. Amplifying the CXCL1/2 signal pathway caused chemoresistance, while CXCR2 blockers augmented the efficacy of chemotherapy against breast tumors, particularly against metastasis [19]. An oncolytic virus armed with a CXCR4 antagonist effectively inhibited the development of spontaneous metastasis and increased overall tumor-free survival [20].

In a previous study, we reported that a peptide antagonist of CXCR4, NT21MP (LGASWHRPDKCCLGYQKRPLP), derived from the residues 1–21 of viral macrophage inflammatory protein II efficiently inhibits SDF-1α-induced proliferation and invasion in breast cancer cells by reducing the levels of phosphorylated AKT and ERK1/2 [21–23]. TGF-β-induced EMT-like activation of the PDGF signaling pathway and the subsequent activation of PI3K in human melanoma cells [24]. Although PDGF signaling is implicated in the TGF-β-mediated epithelial mesenchymal transition of tumor cells, the role of PDGF receptors in the SDF-1α/CXCR4 activation of breast cancer has not been investigated. In the current study, we discussed whether platelet-derived growth factor receptor-α (PDGFRα) is required for SDF-1α/CXCR4 signaling and explored how NT21MP contributes to reversing CXCR4-induced EMT to provide insight into the potential efficacy of NT21MP as adjuvant chemotherapy for breast cancer.

## RESULTS

### The overexpression of PDGF and PDGFR were found in PR cells

**Figure 1:**
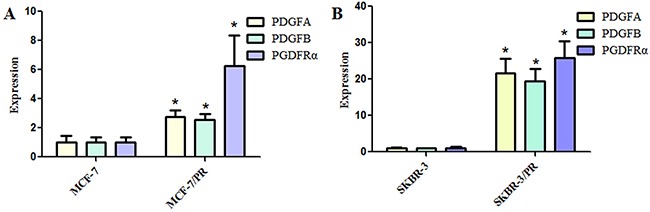
Platelet-derived growth factor and receptor levels in paclitaxel-resistant breast cancer cells **(A)**, Quantitative reverse transcription–polymerase chain reaction (RT-PCR) analysis was used to detect the expressions of platelet-derived growth factor **(A)**, **(B)**, and receptor α (PDGFA, PDGFB, and PDGFRα, respectively) in MCF-7 and MCF-7/PR cells. **(B)**, Quantitative RT-PCR analysis was used to detect the expressions of PDGFA, PDGFB, and PDGFRα in SKBR-3 and SKBR-3/PR cells. *P < 0.05 PR vs control.

Here, we established paclitaxel-resistant breast cancer cells (PR cells) that exhibited resistance to paclitaxel and acquired an EMT feature as described previously [25, 26]. To determine whether PDGF and PDGFR play a critical role in PR-mediated EMT, we measured the expression of PDGFA, PDGFB, and PDGFRα in PR cells and their parental cells. Our results revealed that PDGFA, PDGFB, and PDGFRα were significantly upregulated in PR cells (Figure 1). This finding implicated PDGFRα as a critical mediator of breast cancer oncogenesis, and chemoresistance was associated with EMT in PR cells.

### SDF-1α and CXCR4 expression in breast cancer cells

**Figure 2:**
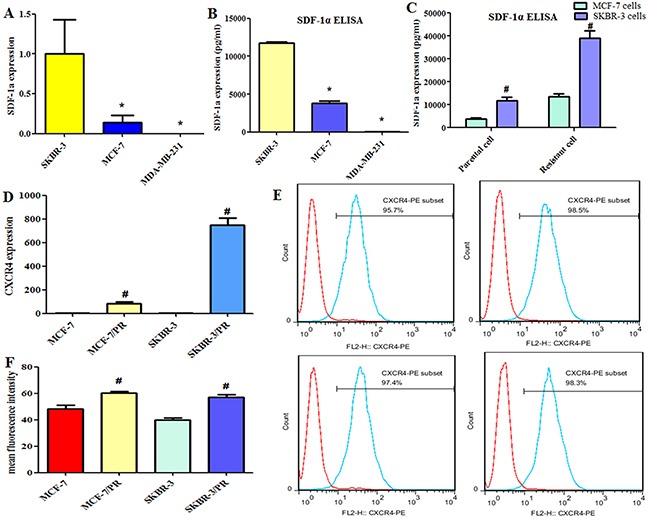
Expression of stromal cell–derived factor 1α (SDF-1α) and CXCR4 in breast cancer cells **(A)**, SDF-1α expression was detected by quantitative reverse transcription–polymerase chain reaction in SKBR-3, MCF-7, and MDA-MB-231 cells. **(B)**, ELISA was used to measure the levels of SDF-1α secreted by the SKBR-3, MCF-7, and MDA-MB-231 cells. **(C)**, ELISA was used to measure the levels of SDF-1α secreted in parental and resistant cells. **(D)**, The CXCR4 level in MCF-7, MCF-7 PR, SKBR-3, and SKBR-3 PR cells analyzed by qRT-PCR. **(E)**, The CXCR4 level in these cells analyzed by flow cytometry using antibody for CXCR4. **(F)**, The mean fluorescence intensity of CXCR4 level analyzed by flow cytometry. *P < 0.05 vs SKBR-3 cells, ^#^P < 0.05 PR vs control.

The expression of SDF-1α was examined in SKBR-3, MCF-7 and MDA-MB-231 cells by quantitative PCR and enzyme-linked immunosorbent assay (ELISA). Our results illustrated a cell-dependent and resistance-dependent gene expression spectrum for the SDF-1α gene (Figure 2A, 2B, 2C) with high expression levels in SKBR-3 cells (11.8 ng/mL) and resistant SKBR-3 PR cells (39.1 ng/mL) as well as in MCF-7 PR cells (13.5 ng/mL vs 3.8 ng/mL in MCF-7 cells). In MDA-MB-231 cells, SDF-1α showed relatively low expression levels (28.9 pg/mL). We used quantitative RT-PCR and flow cytometry to measure the levels of CXCR4 in PR cells and their parental cells and found that CXCR4 was significantly upregulated in MCF-7 PR and SKBR-3 PR cells compared with their parental cells (Figure 2D, 2E, 2F).

### Effect of NT21MP on paclitaxel resistance

**Figure 3:**
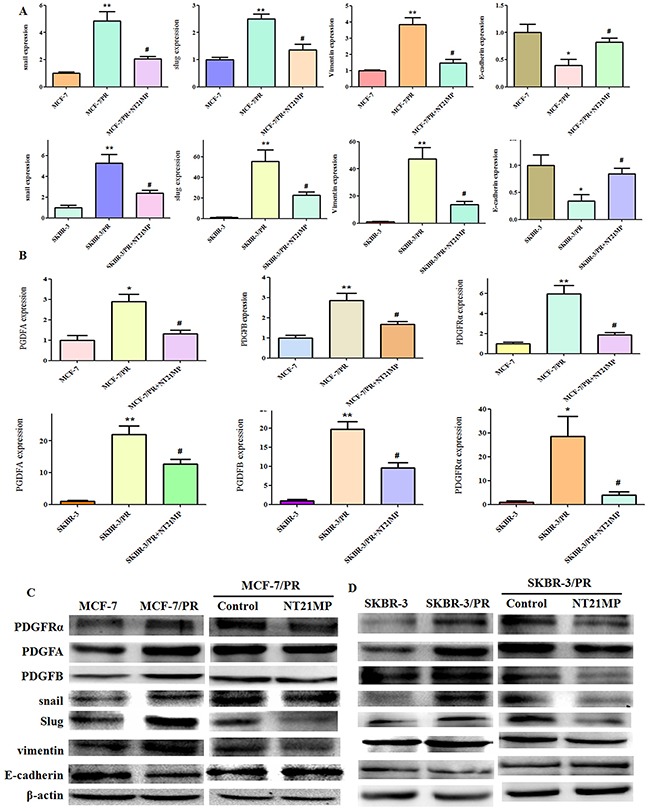
Effect of NT21MP on MCF-7 and SKBR-3 cell drug resistance **(A)**, Quantitative RT-PCR assays were conducted to detect the expression of resistant markers in parental and PR cells and the NT21MP treatment group. **(B)**, Quantitative PCR assays were conducted to detect the expression of PDGFA, PDGFB, and PDGFRα in parental and PR cells and the NT21MP treatment group. **(C)**, Western blotting results for the expression of resistant markers in MCF-7 and MCF-7 PR cells and the NT21MP treatment group. **(D)**, Western blotting results for the expression of resistant markers in SKBR-3 and SKBR-3 PR cells and the NT21MP treatment group. *P < 0.05 and **P < 0.01 PR vs control and #P < 0.05 NT21MP treatment vs control in PR cells.

To further investigate whether NT21MP could reverse drug resistance in paclitaxel-resistant cells, we added 1 μg/mL NT21MP to MCF-7 PR and SKBR-3 PR cells. We found that NT21MP significantly inhibited the expressions of Snail, Slug, Vimentin, PDGFA, PDGFB, and PDGFRα but increased E-cadherin expression (Figure 3A, 3B, 3C, 3D), suggesting that NT21MP partly reversed the phenotype of EMT in PR cells.

### NT21MP inhibits biological activity in resistant cells

**Figure 4:**
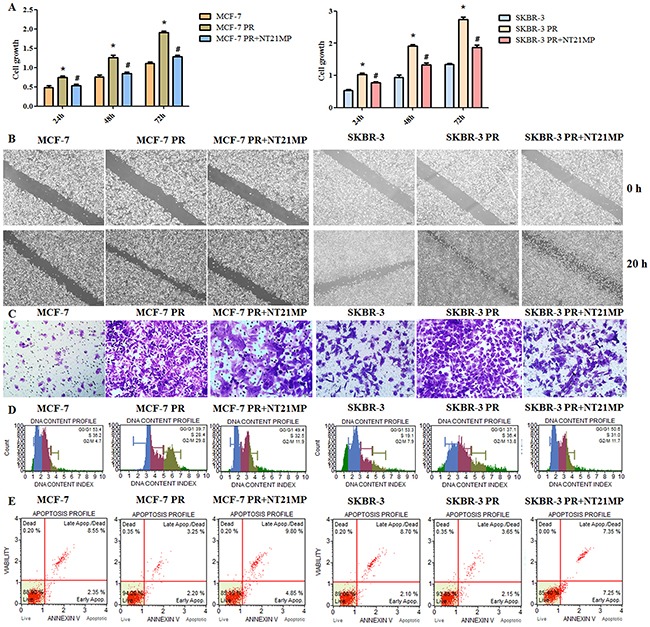
Effect of NT21MP on PR cell biological activity **(A)**, Sulforhodamine B assays were performed to measure the proliferation in PR cells treated with NT21MP. **(B)**, Wound healing assays were used to detect the motility in PR cells treated with NT21MP. **(C)**, Invasion assays were conducted in PR cells treated with NT21MP. **(D)**, Flow cytometry was used to evaluate the cell cycles of PR cells after treatment with NT21MP. **(E)**, Apoptosis was detected in PR cells after treatment with 1 μg/mL NT21MP. *P < 0.05, PR vs control; ^#^P < 0.05, NT21MP treatment vs control in PR cells.

To further validate the role of NT21MP in cell resistance, we examined the proliferation, motility, invasion, cycle, and apoptosis capacities of paclitaxel-resistant cells after 1 μg/mL NT21MP treatment. Our results showed that NT21MP attenuated cell proliferation, motility and invasion capacities in PR cells (Figure 4A, 4B, 4C). A cell cycle analysis revealed a reduced G_0_/G_1_ phase in PR cells and that NT21MP reversed the change from 39.7% to 49.4% in MCF-7 PR cells and from 37.1% to 50.0% in SKBR-3 PR (Figure 4D), demonstrating that NT21MP could cause cell cycle arrest in PR cells. We found lower percentages of apoptotic cells in PR cells compared with the parental cells and that NT21MP treatment increased the percentage (Figure 4E).

### Construction of over or underexpressing CXCR4 breast cancer cell lines

**Figure 5:**
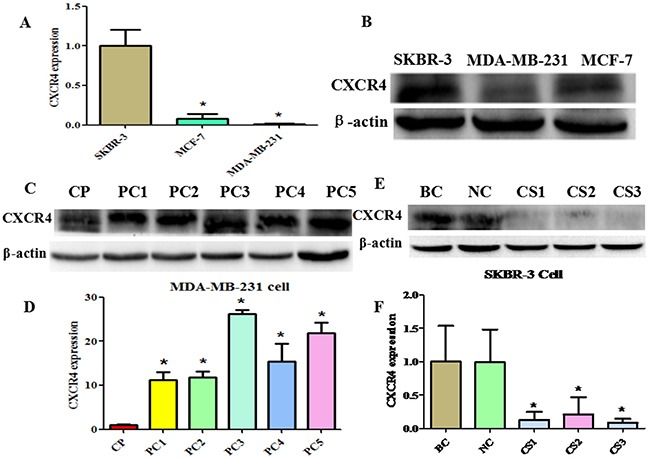
Construction of CXCR4 over- and underexpressing breast cancer cell lines **(A)** and **(B)**, Quantitative reverse transcription–polymerase chain reaction (RT-PCR) and western blotting were conducted to measure CXCR4 expression in SKBR-3, MCF-7, and MDA-MB-231 cells, respectively. *P < 0.05 vs SKBR-3 cells. **(C)**, Western blotting analysis was performed to detect the expression of CXCR4 in MDA-MB-231 cells treated with CXCR4-overexpressing (pcDNA-CXCR4). CP: control pcDNA-CXCR4; PC1-5: pcDNA-CXCR4 1-5. **(D)**, Quantitative results are illustrated for panel C. *P < 0.05 vs control. **(E)**, Western blotting analysis was performed to detect the expression of CXCR4 in SKBR-3 cells treated with CXCR4 siRNA. BC: blank control siRNA; NC: negative siRNA; CS1-3: CXCR4 siRNA 1-3. **(F)**, Quantitative results are illustrated for panel E. *P < 0.05 vs control.

NT21MP has been demonstrated to exert its function via binding CXCR4 [27]. Therefore, to obtain further more insight into the mechanism of NT21MP by blocking CXCR4 signal pathways in reverse drug resistance, we depleted or increased the expression of CXCR4 in the parental cells. As demonstrated in Figure 5A, 5B, SKBR-3 cells demonstrated the highest levels of CXCR4, while MDA-MB-231 demonstrated the lowest levels. Therefore, we used MDA-MB-231 cells to establish the pcDNA-CXCR4-MDA-MB-231 cells (PC-MDA-MB-231 cells) that express higher CXCR4 levels than the parental cells and successfully established stable CXCR4-overexpressing cells (Figure 5C, 5D). CXCR4 siRNA transfection significantly inhibited CXCR4 expression in SKBR-3 cells (CS-SKBR-3 cells) after CXCR4 siRNA transfection (Figure 5E, 5F).

### CXCR4/PDGFRα signaling pathway plays a role in reversing drug resistance by NT21MP

**Figure 6:**
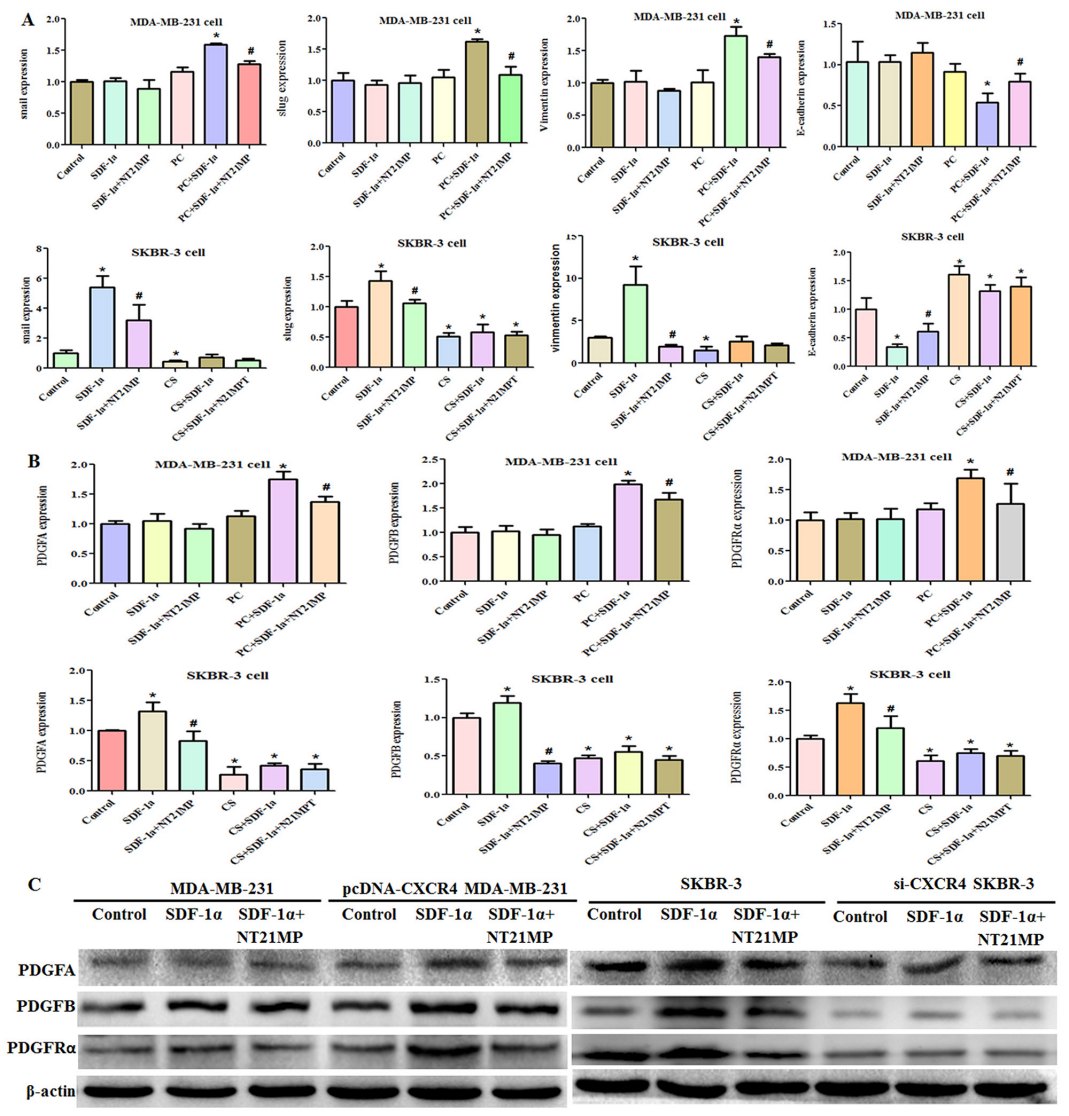
NT21MP regulates drug resistance via CXCR4 **(A)**, Quantitative RT-PCR assays were conducted to detect the expression of EMT markers in MDA-MB-231 and PC-MDA-MB-231 cells or in SKBR-3 and CS-SKBR-3 cells with CXCR4 siRNA after 100 ng/mL SDF-1α treatment alone or combined with 1 μg/mL NT21MP. **(B)**, Quantitative RT-PCR assays were conducted to detect the expressions of PDGFA, PDGFB, and PDGFRα for panel A. *P < 0.05, SDF-1α, CXCR4-overexpressing, and si-CXCR4 group vs control; ^#^P < 0.05, NT21MP treatment vs SDF-1α group. **(C)**, Western blotting results for panel B.

To further investigate whether CXCR4 has a critical role in the ability of NT21MP to reverse drug resistance, we measured the expression of EMT markers after 100 ng/mL SDF-1α treatment alone or combined with 1 μg/mL NT21MP. The addition of SDF-1α did not remarkably induce a change in Snail, Slug, Vimentin, E-cadherin, PDGFA, PDGFB, or PDGFRα expression in the MDA-MB-231 cells, and NT21MP did not inhibit their expression. In the PC-MDA-MB-231 cells, the levels of Snail, Slug, Vimentin, PDGFA, PDGFB, and PDGFRα were increased, while that of E-cadherin was decreased after SDF-1α treatment; however, NT21MP could reverse these changes. Similarly, we found that SDF-1α treatment led to increased expression of Snail, Slug, Vimentin, PDGFA, PDGFB, and PDGFRα and decreased E-cadherin in SKBR-3 cells. NT21MP attenuated the effect induced by SDF-1α. These changes were attenuated when CXCR4 expression was interfered with compared to the matching group in SKBR-3 cells regardless of SDF-1α or NT21MP treatment (Figure 6A, 6B, 6C). Taken together, these results indicated that CXCR4 played a critical role in NT21MP-reversed drug resistance and established a link between CXCR4 and the PDGFRα signaling.

### Effect of CXCR4 on cell proliferation and migration abilities

**Figure 7:**
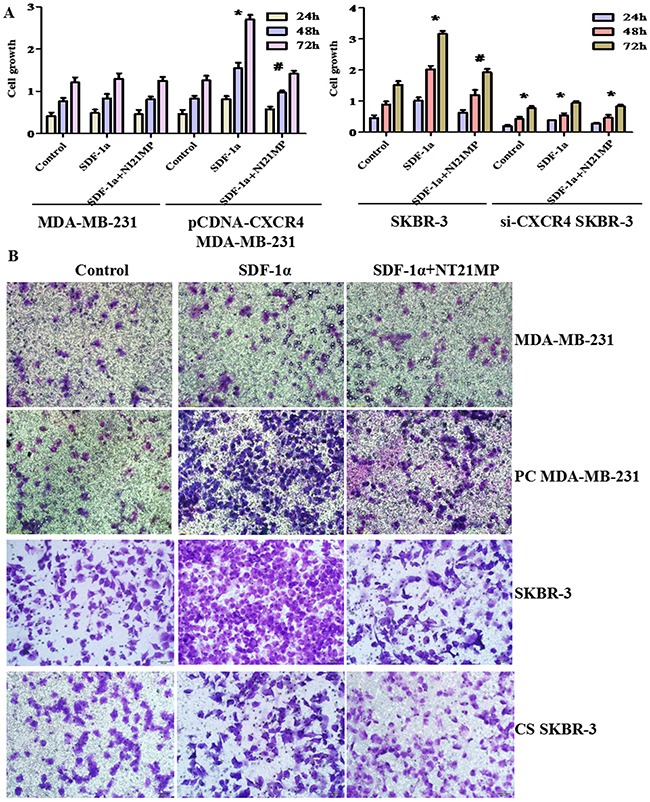
Effect of CXCR4 on cell proliferation and migration abilities **(A)**, Sulforhodamine B (SRB) assays were conducted to detect the proliferation capacities in MDA-MB-231 and PC-MDA-MB-231 cells or SKBR-3 and CS-SKBR-3 cells after treatment with 100 ng/mL SDF-1α alone or combined with 1 μg/mL NT21MP. **(B)**, Invasion assays were conducted in MDA-MB-231 and PC-MDA-MB-231 cells or SKBR-3 and CS-SKBR-3 cells after SDF-1α treatment alone or combined with NT21MP. PC MDA-MB-231: CXCR4 over-expression in MDA-MB-231 cells and CS-SKBR-3: CXCR4 siRNA in SKBR-3 cells. *P < 0.05, SDF-1α, CXCR4-overexpressing, and si-CXCR4 group vs control; ^#^P < 0.05, NT21MP treatment vs SDF-1α group.

To confirm that NT21MP reverses EMT via a CXCR4-dependent mechanism, we used Sulforhodamine B (SRB) and chemotaxis assays to investigate the effect of CXCR4 on cell proliferation and cell migration ability in MDA-MB-231, PC-MDA-MB-231, SKBR-3, and CS-SKBR-3 cells. These cells were treated with 100 ng/mL SDF-1α alone or combined with 1 μg/mL NT21MP for 24, 48, or 72 h. The chemotaxis assays methods as noted above and cell grouping were the same as the SRB assays. The addition of SDF-1α did not remarkably promote cell proliferation or migration in the MDA-MB-231 cells, and NT21MP did not inhibit it (Figure 7A, 7B). The proliferation and migration capacities of the PC-MDA-MB-231 cells were increased by SDF-1α but were markedly inhibited by NT21MP. Similarly, we found that SDF-1α promoted the proliferation and migration capacities of SKBR-3 cells and that NT21MP attenuated these capacities induced by SDF-1α treatment (Figure 7A, 7B). However, there were lower growth and migration capacities in SKBR-3 cells with CXCR4 siRNA treatment than the matching group. These results showed that CXCR4 played an important role in breast cancer cell proliferation and migration.

### NT21MP reverses drug resistance via PDGF signaling pathway

**Figure 8:**
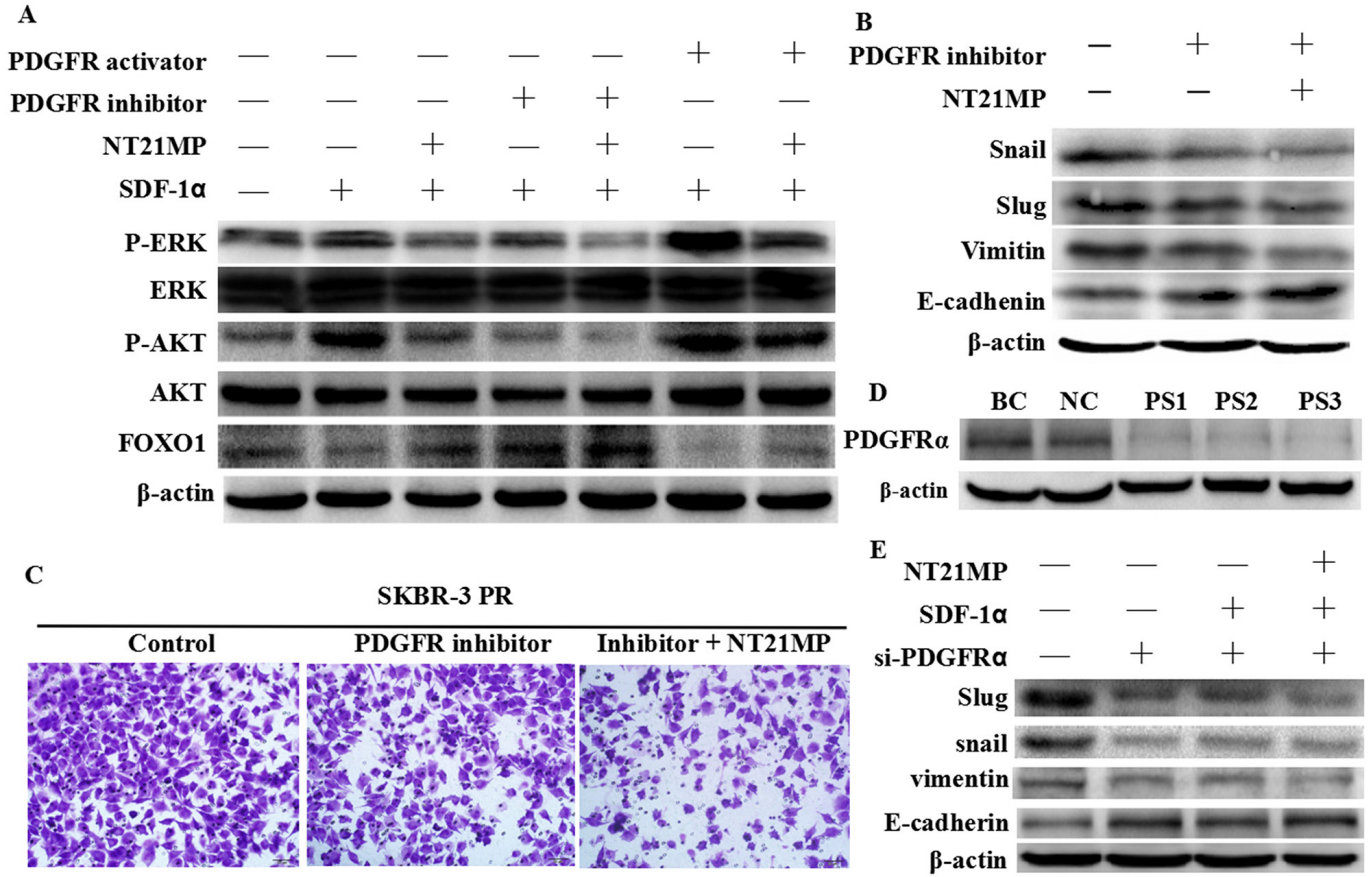
Inhibition of PDGFRα enhances the effect of NT21MP-reversed EMT and invasion in SKBR-3 PR cells **(A)**, Western blot assays were used to detect signal pathway molecule expression. In each group, the cells were treated after SDF-1α treatment alone or combined with NT21MP or PDGFRα inhibitor or activator in SKBR-3 PR cells. **(B)**, Western blotting analysis was performed to detect the expressions of EMT markers in SKBR-3 PR cells treated with PDGFRα inhibitor alone or combined with NT21MP. **(C)**, Invasion assays were conducted in SKBR-3 PR cells treated with PDGFRα inhibitor alone or combined with NT21MP. **(D)**, Western blotting analysis was performed to detect the expression of PDGFRα in SKBR-3 PR cells treated with PDGFRα siRNA. BC: blank control siRNA; NC: negative siRNA; PS1-3: PDGFRα siRNA 1-3. **(E)**, Western blotting analysis was performed to detect the expression of EMT markers in SKBR-3 PR cells treated with PDGFRα siRNA alone or combined with NT21MP.

To further investigate the signaling pathway by which NT21MP reverses drug resistance, we used western blotting to analyze signal protein expression after SDF-1α treatment alone or combined with NT21MP, PDGFR inhibitor, or PDGFR activator. SDF-1α induced the upregulation of PDGFA, PDGFB, PDGFRα expression and phosphorylation of AKT and ERK1/2, a downstream target of PDGF signaling, and inhibited the expression of FOXO1, a downstream target of PI3K signaling. The inhibition of PDGFRα by PDGFRα inhibitors and NT21MP attenuated the SDF-1α-stimulated phosphorylation of AKT and ERK1/2 and breast cancer cell migration and had a synergistic effect compared with single treatment with either NT21MP or PDGFR inhibitor (Figure 8A, 8C). However, the PDGFR activator induced a reverse change that had a synergistic effect, with SDF-1α and NT21MP cancelling out the effect of the PDGFR activator. To determine whether the antitumor activity of NT21MP is due to the inhibition of PDGFRα target genes, we detected the expression of EMT markers and the invasive activity in PR cells after PDGFRα siRNA transfection or PDGFRα inhibitor treatment combined with NT21MP. A PDGFRα inhibitor could inhibit the expressions of Snail, Slug and Vimentin but increased E-cadherin expression (Figure 8B), and the combination group showed a more pronounced change. Similarly, the PDGFRα inhibitor could inhibit the migration capacities of SKBR-3 PR cells and showed a more pronounced effect in the combination group with NT21MP (Figure 8C). Moreover, our results showed that the depletion of PDGFRα increased E-cadherin protein levels and decreased the expression of mesenchymal markers including Snail, Slug, and Vimentin; however, the addition of SDF-1α did not remarkably induce a change and NT21MP did not inhibit it (Figure 8E). Taken together, these results indicate that PDGFRα plays a critical role in reversing PR-induced EMT in breast cancer cells through NT21MP.

### NT21MP increases the paclitaxel sensitivity of PR cells

**Figure 9:**
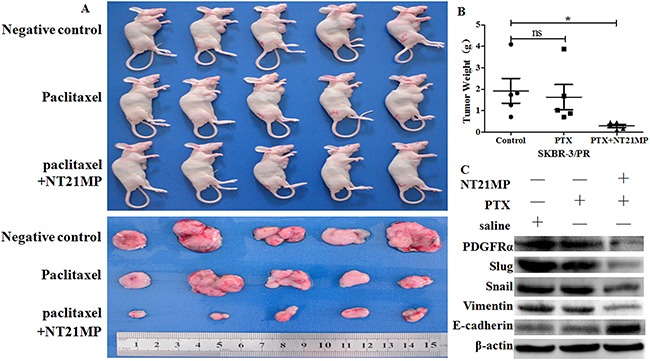
NT21MP enhances PR cells to paclitaxel sensitivity *in vivo* **(A)**, Photographs of tumor size at the time of euthanization in which NT21MP retarded the growth of SKBR-3 PR cells in mice. **(B)**, Total tumor weights in mice were measured at the time of euthanization. *P < 0.05, NT21MP group vs control. **(C)**, Western blotting analysis was performed to detect the expression of PDGFRα and EMT markers.

To determine whether NT21MP increases the paclitaxel sensitivity of PR cells *in vivo*, we established a SKBR-3 PR cells breast cancer mouse model. As shown in Figure 9A, 9B, NT21MP treatment significantly inhibited tumor growth compared with paclitaxel and a negative control. Notably, we observed that the inhibition of tumor EMT by NT21MP correlates with the decreased expression of PDGFRα in the NT21MP-treated tumors compared with paclitaxel and the negative control (Figure 9C). This data strongly suggest that CXCR4/PDGFRα is a potential target of NT21MP in the inhibition of the EMT process in breast cancer and demonstrate that PR cells with NT21MP treatment were significantly more sensitive to paclitaxel-induced cell growth inhibition.

## DISCUSSION

The EMT is associated with tumorigenesis and metastasis as well as chemoresistance of cancer. Therefore, elucidating the mechanisms that govern the acquisition of EMT would likely be useful for devising targeted therapeutic approaches to overcome or prevent resistance to conventional cancer therapeutics. Paclitaxel promotes the polymerization of tubulin, thereby causing cell death by disrupting the normal microtubule dynamics [28] and is highly clinically responsive to chemotherapies. Recent studies have shown that links between paclitaxel resistance and EMT lead breast cancer patients to develop chemoresistance [29, 30]. In our previous studies, we showed that paclitaxel-resistant cells (SKBR-3 PR and MCF-7 PR) gained mobility and invasiveness through the acquired EMT feature, as described previously [25, 26], and observed the co-overexpression of PDGFA, PDGFB, PDGFRα, CXCR4, and SDF-1α compared to their parental cells.

CXCR4 expression is higher in dedifferentiated cells than in normal cells and independently predicted poor survival in tumors [31]. The activation of molecular pathways, such as Akt and ERK, induces tumor progression and is associated with CXCR4 expression. Agents specifically directed against the CXCR4 receptor could compete with the SDF-1α ligand to inhibit cellular function [32, 33]. By blocking the receptor with NT21MP, which is a selective synthetic polypeptide derived from vMIP-II, from interacting with its natural ligand, our previous study achieved the inhibition of primary tumor growth and metastasis [22, 23].

CXCR4 is also a key regulator of the EMT process, through which it could activate signals associated with tumor progression [34]. The targeted silencing of CXCR4 inhibits epithelial-mesenchymal transition in oral squamous cell carcinoma [35]. Blockade of SDF-1α/CXCR4 signaling inhibits the expression of kisspeptin-10 (KP-10) and mesenchymal markers N-cadherin and Vimentin [36]. Additionally, miR-381 inhibited breast cancer cells proliferation, EMT and metastasis by targeting CXCR4 [37]. Consistently, Nef-M1 peptide, a peptide antagonist of CXCR4, inhibits tumor EMT process by targeting CXCR4 [38]. In line with these findings, our present work demonstrated that NT21MP also reverses breast cancer EMT via a CXCR4/PDGFRα-dependent mechanism both *in vitro* and *in vivo*.

First, our study demonstrated a significant association between CXCR4 and paclitaxel resistance and EMT. The expression of CXCR4 in PR cells has been found to be associated with PDGFRα and the induction of EMT. We used a breast cancer cell line that does not express CXCR4 to determine whether NT21MP inhibits the EMT process through the CXCR4/PDGFRα signaling pathway and demonstrated that NT21MP targeted CXCR4, inhibits EMT, and inhibits tumor progression. Notably, the CXCR4-expressing cells became vulnerable to the NT21MP-induced inhibition of drug resistance gene expression. Furthermore, cells that express CXCR4 became susceptible to the shift from a mesenchymal to epithelial profile in response to NT21MP.

The PI3K/Akt signaling pathway can affect the EMT to influence tumor aggressiveness [39]. TGFβ induced an EMT-like effect on the activation of the PDGF signaling pathway and the subsequent activation of PI3K in human melanoma cells [40], and CXCR4 signaling induced EMT by the PI3K/AKT and ERK pathways in glioblastoma [41]. Receptor tyrosine kinases (RTKs), such as PDGFRα (platelet-derived growth factor receptor-α), contributes to EMT maintenance by activating STAT1, PI3K/AKT, MAPK, and other distinct pathways and confers a more invasive and drug-resistant phenotype [42–44].

Here, we report an interaction between CXCR4 and PDGFRα in promoting chemoresistance in breast cancer cells. Platelet-derived growth factor (PDGF) regulates angiogenesis through CXCR4-mediated signaling [45] as a pro-angiogenic growth factor. Thus, targeting CXCR4 to inhibit the PDGFRα signaling pathway with an appropriate therapeutic agent may represent a means of controlling the breast cancer progression. We demonstrate here that CXCR4 signaling, which increases PDGFRα activity, could enhance tumor invasion ability. This study showed that the expression of PDGFRα in PR cells is associated with an increased expression of CXCR4. The inhibitory role of NT21MP on tumor resistance and the expression of CXCR4 and PDGFRα also demonstrated a correlation between the decreased expression CXCR4 and PDGFRα and the inhibition of tumor resistance *in vitro* and *vivo*. The blockade of PDGFR, a key mediator of CXCR4 signaling, similarly reversed EMT markers and cell invasion. These results showed cross-talk of CXCR4 and PDGF signaling in human breast cancer, resulting in the epithelial-to-mesenchymal transition in a PDGFRα-dependent manner. *In vivo*, tumor growth was assessed by the subcutaneous inoculation of cells into BALB/c nude mice. PDGFRα and EMT markers were detected in the tumor tissues derived from mice. NT21MP inhibited tumor growth and the expression of PDGFRα, and EMT-associated proteins had similar expression patterns to the experimental results observed *in vitro*, suggesting NT21MP may inhibit tumor resistance by inhibiting the CXCR4/PDGFRα signaling mechanism in breast cancer.

In summary, our findings confirmed that SDF-1α can activate PI3K/AKT and ERK1/2 via the PDGFR signaling pathway combined with CXCR4 and play a key role in inducing chemoresistance. NT21MP can reverse drug resistance by inactivating PDGFRα and subsequently inhibiting the PI3K/AKT and ERK1/2 signaling pathways by blocking the SDF-1-CXCR4 axis. We also identified PDGFRα as an effector that mediates CXCR4-induced EMT, suggesting that targeting the CXCR4-PDGFRα-PI3K pathway may be beneficial to NT21MP-reversed drug resistance in breast cancer.

## MATERIALS AND METHODS

### Cell culture

The human breast cancer cell lines MCF-7, SKBR-3, and MDA-MB-231 were cultured in Dulbecco's modified Eagle medium (Invitrogen, Carlsbad, CA, USA) supplemented with 10% fetal bovine serum (FBS) and maintained in a humidified 5% CO_2_ incubator at 37˚C.

### Reagents and antibodies

Primary antibodies against Snail, Slug, Vimentin, E-cadherin, PDGFA, PDGFB, PDGFRα, P-AKT, AKT, P-ERK, ERK, CXCR4 and β-actin were obtained from Santa Cruz Biotechnology (Santa Cruz, CA, USA). Anti-FOXO1 antibodies were purchased from Abcam (Cambridge, MA, USA). All secondary antibodies were obtained from Pierce (Rockford, IL, USA). Lipofectamine 2000 was obtained from Invitrogen. SRB was purchased from Sigma (St. Louis, MO, USA).

### Quantitative reverse transcription–polymerase chain reaction analysis of gene expression

The total RNA from treated cells was isolated with Trizol (Invitrogen) according to the manufacturer's protocols and then reverse-transcribed into cDNA by a RevertAid First Strand cDNA Synthesis Kit (Thermo Scientific, USA). The relative quantitative real-time reverse transcription–polymerase chain reaction (RT-PCR) procedures were performed as described previously [26]. The primers used in PCR reaction are Table 1.

**Table 1:** The primers used in PCR reaction

Name	Forward primer	Reverse primer
PDGFA	CAAGACCAGGACGGTCATTT	CCTGACGTATTCCACCTTGG
PDGFB	TCCCGAGGAGCTTTATGAGA	GGGTCATGTTCAGGTCCAAC
PDGFRα	GAAGCTGTCAACCTGCATGA	CTTCCTTAGCACGGATCAGC
Snail	CGGAAGCCTAACTACAGCGA	GGACAGAGTCCCAGATGAGC
Slug	CATGCCTGTCATACCACAAC	GGTGTCAGATGGAGGAGGG
Vimentin	TGTCCAAATCGATGTGGATGTTTC	TTGTACCATTCTTCTGCCTCCTG
E-cadherin	GAAGTGTCCGAGGACTTTGG	CAGTGTCTCTCCAAATCCGATA
SDF-1α	CCGCGCTCTGCCTCAGCGACGGGAAG	CTTGTTTAAAGCTTTCTCCAGGTACT
CXCR4	GAACCCTGTTTCCG GAAGA	CTTGTCCGTCATGCTTCTCA
GAPDH	CAGCCTCAAGATCATCAGCA	TGTGGTCATGAGTCCTTCCA

### Western blotting analysis

The treated cells were washed with phosphate-buffered saline (PBS) and disrupted in RIPA buffer mixed with phenylmethanesulfonyl fluoride and phosphatase inhibitor cocktails (Sigma) on ice. The protein concentrations were measured using a BCA Protein Assay kit (Pierce). The protein was then denatured with sample buffer (X5) for 10 minutes at 99˚C. The equivalent proteins were separated on a sodium dodecyl sulfate–polyacrylamide gel and then transferred onto polyvinylidene fluoride (PVDF) membranes (Bio-Rad, Hercules, CA, USA). The PVDF membranes were blocked with 5% bovine serum albumin–Tris-buffered saline and Tween 20 (TBST) for 2 h at room temperature and then incubated with the indicated primary antibodies overnight at 4˚C. After being washed with TBST, the membranes were incubated with secondary antibody for 2 h at 37˚C. The blots were scanned by the Gel Image Analysis System (Bio-Rad).

### Enzyme-linked immunosorbent assay

The supernatants were collected after centrifugation, and the levels of SDF-α secreted by the SKBR-3, MCF-7, and MDA-MB-231 cells were quantified using an enzyme-linked immunosorbent assay (ELISA; ExCell Biology, Shanghai, China) according to the manufacturer's instructions.

### The CXCR4 level of parent and paclitaxel-resistant cells

The MCF-7, MCF-7/PR, SKBR-3, and SKBR-3/PR cells of × 10^5^ were incubated with mouse monoclonal anti-human CXCR4 antibody (12G5; R&D Systems, Minneapolis, MN) in BSA/PBS for 1 h at 4˚C and then treated with goat anti-mouse-IgG-FITC (BioLegend, San Diego, CA). Resulting cells were subjected to C6 Accuri flow cytometer (Accuri Cytometers, Ann Arbor, MI), and acquired data were analyzed by FlowJo.

### Cell proliferation assay

The MCF-7, MCF-7/PR, SKBR-3, and SKBR-3/PR cells (5 × 10^3^) were seeded in each well of the 96-well plates overnight. The cells were then treated with 1 μg/mL NT21MP for 24, 48, or 72 h. The SRB assay was performed as described previously [27].

### Wound healing assay

The cells were seeded into six-well plates. After the cells reached 90% confluence, a wound was created using a 10 μL pipette tip, and the detached cells were removed by washing with PBS. Then, some of the MCF-7/PR, SKBR-3/PR cells were treated with 1 μg/mL NT21MP for 20 h. Images were collected at 0 and 20 h.

### Cell invasion assay

The invasive activity of paclitaxel-resistant breast cancer cells treated with NT21MP or PDGFR inhibitor was evaluated using Transwell inserts as described previously [26]. 600 μL serum-free medium with SDF-1α (100 ng/mL, R&D Systems, Minneapolis, MN) was added to the bottom wells of the 24-well plate, and no SDF-1α was used in the blank group. One million SKBR-3 cells resuspended in buffer with NT21MP or PDGFR inhibitor were added to the upper chamber insert. After 24 h, the cells in the upper chamber had partly invaded the underside. The invaded cells were fixed with 4% paraformaldehyde, stained with Giemsa solution, and photographed under a microscope.

### Cell cycle analysis

Breast cancer cells were plated at 1 × 10^5^ cells/well on six-well plates and treated with 1 μg/mL NT21MP. Cells were incubated for 48 h, then washed twice with cold PBS and fixed in 70% ethanol at 4˚C overnight. The cells were then re-suspended at 1 × 10^6^ cells/mL in PBS and incubated with 100 μg/mL RNase A and 50 mg/mL propidium iodide at room temperature for 30 min. The distribution of the cells throughout the cell cycle was determined using flow cytometry.

### Cell apoptosis assay

Breast cancer cells were seeded at a density of 1 × 10^5^ cells/well on six-well plates and treated with 1 μg/mL NT21MP. After 48 h, the cells were washed in PBS, re-suspended in 1% FBS, and incubated with 100 μL of Muse Annexin V & Dead Cell Reagent (Muse, les, China) for 20 min in the dark. We then analyzed the results using flow cytometry.

### Establishment of stable transfected cell lines

The pcDNA3.1-CXCR4 system containing Geneticin (G418; Sigma) as a selective marker was constructed by GenePharma (Suzhou, China). MDA-MB-231 cells were transfected with Lipofectamine™ 2000 reagent. After 48 hours of transfection, the cells were exposed to growth medium containing G418 800 μg/mL (the minimum concentration needed to kill this cell line). After 14 days, G418-resistant clones were randomly collected. After more than 14 days, cells that grew under 500 μg/mL G418 treatment likely included a copy of the G418-resistant gene in their genome and were identified as stable transfected cell lines or pcDNA-CXCR4-MDA-MB-231 cells. Non-transfected MDA-MB-231 cells were used as a negative control.

### Transfection

Cells were seeded in six-well plates and transfected with CXCR4 siRNA or control siRNA using Lipofectamine 2000 as described previously [26]. The sequences used for CXCR4 and PDGFRα siRNA are as Table 2. After incubation, the cells were analyzed further as described in the Results section.

**Table 2:** The sequences of CXCR4 and PDGFRα siRNA

Name	Sense	Antisense
CXCR4 siRNA1	GAAGCAUGACGGACAAGUAdTdT	dTdTCUUCGUACUGCCUGUUCAU
CXCR4 siRNA2	GGAAGCUGUUGGCUGAAAAdTdT	dTdTCCUUCGACAACCGACUUUU
CXCR4 siRNA3	CUGUCCUGCUAUUGCAUUAdTdT	dTdTGACAGGACGAUAACGUAAU
PDGFRα siRNA1	GCAGGCACAUUUACAUCUATT	UAGAUGUAAAUGUGCCUGCTT
PDGFRα siRNA2	CCGAGACUCCUGUAACCUUTT	AAGGUUACAGGAGUCUCGGTT
PDGFRα siRNA3	GGAAGAAGACAGUGGCCAUTT	AUGGCCACUGUCUUCUUCCTT

### Animal experiments

Female nude mice (4-6 weeks, 18-20 g) were purchased from Shanghai SLAC Laboratory Animal Co. Ltd (Shanghai, China). The mice were housed and maintained in the Experimental Animal Center of Bengbu Medical College under specific pathogen-free conditions. All animal experiments were used in accordance with Animal Care and Use Guidelines of Bengbu Medical College, and the protocol was approved by the Committee on the Ethics of Animal Experiments of Bengbu Medical College Institutional Users of Animal Care Committee. The animals were acclimatized to laboratory conditions for 1 week prior to the experiments. SKBR-3 PR cells (5×10^6^) were injected in the second right mammary gland, respectively. When the xenograft volumes reached 15 mm^3^, all mice were divided into 3 groups (n = 5 mice each), including a control group (saline group), PTX group, and combination group (PTX + NT21MP). Then, the mice were treated with PTX (15 mg/kg), saline twice per week or NT21MP (500 μg/kg) 5 days per week for 4 weeks with 100 μL per mouse. PTX or saline was injected intraperitoneally, and NT21MP was injected through the tail vein. At the end of the experiment, the mice were sacrificed and tumors were dissected for weighing and western blot analysis.

### Histologic sections and immunohistochemistry

Immunohistochemical studies were performed according to our group's previous methods [24]. Tumor proliferation was assessed by PCNA staining, and tumor apoptosis was evaluated by TUNEL as described previously [24].

### Statistical analysis

Statistical comparisons between two different groups were determined using an unpaired Student's t-test in GraphPad Prism 5.0 (GraphPad Software, La Jolla, CA, USA). The results are presented as the mean ± standard deviation (SD). Values of P < 0.05 are considered statistically significant.
